# Psychiatry outpatient visits by atopic dermatitis patients varying in the complexity of their prescriptions: A nationwide cohort study conducted from 2005 to 2013

**DOI:** 10.1097/MD.0000000000005411

**Published:** 2016-12-09

**Authors:** Jaeyong Shin, Young Choi, Eun-Cheol Park, Kwang Hoon Lee, Seo Young Hwang, Sang Ho Oh, Sang Gyu Lee

**Affiliations:** a Department of Preventive Medicine; b Institute of Health Services Research, College of Medicine; c Department of Public Health, Graduate School; d Department of Dermatology, Severance hospital, College of medicine; e Department of Hospital Management, Graduate School of Public Health, Yonsei University, Seoul, Korea.

**Keywords:** atopic dermatitis, complexity, medication, prescription, psychiatric disorder, psychological stress

## Abstract

The uncomfortable symptoms of atopic dermatitis (AD) often trigger psychological stress.

We investigated the association between AD severity and outpatient visits to psychiatrists using Korean National Health Insurance claims data from 2004 to 2013.

This was a retrospective cohort analysis; we estimated hazard ratios (HRs).

Of the 266,182 subjects, 18,290 (6.9%) had a history of visiting a psychiatrist, and 25,419 (9.5%) were diagnosed with AD. Compared with the subjects without AD, subjects with AD had a higher HR for visiting a psychiatrist (3.70, 95% confidence interval [CI]:48 3.43–3.98), and this significantly increased as the complexity of the AD medications increased. Subjects who received topical treatments plus a systematic steroid (HR: 4.88, 95% CI: 4.51–5.27) and those who received topical treatments plus a systematic steroid plus a systematic calcineurin inhibitor (HR: 9.56, 95% CI: 4.29–21.28) had higher HRs compared with those without AD.

Severe AD patients exhibited greater psychological stress than those without AD or with mild AD symptoms. Moreover, medication diversity may represent an efficient and low-cost method by which to identify a vulnerable population.

## Abstract 2

Supplemental Digital Content is available in the text

## 1 Introduction

Atopic dermatitis (AD) is a chronic inflammatory skin disorder that causes itching.^[1,2]^ This pruritic skin disorder typically occurs during childhood or early puberty but may persist throughout one's lifetime with a waxing and waning clinical course. Since the 1960s, there has been a 3-fold increase in the prevalence of AD^[3]^ and, thus, this chronic inflammatory disorder of the skin has become a major global public health concern. In the United States, Northern and Western Europe, Japan, Korea, and Australia, 10–20% of children are thought to have AD.^[4]^ In Korea, the prevalence of AD in children younger than 24 months increased from 19.8% to 23.8% between 2003 and 2008,^[5]^ and its incidence in the general Korean general population aged 19 years and older is 7.1%.^[6]^

Because AD is usually accompanied by annoying itching and discomfort during routine everyday life, it is an emerging social concern in many developed countries.^[7]^ Furthermore, these types of uncomfortable symptoms are often associated with psychological stress and impaired achievement at school.^[8]^ Therefore, it is important to assess the levels of psychological stress in AD patients and to develop methods to prevent psychological and physical problems. However, it is difficult to measure the increased risk of psychological burden among AD patients using population-based approaches. Thus, the present study attempted to investigate the relationship between increased severity of AD and psychological stress and burden using national claims data for 2002 to 2013. Additionally, this study sought to identify the age groups and specific types of psychological disorder that are especially likely to be associated with a history of AD.

## 2 Materials and methods

### 2.1 Data

We analyzed the Korea National Health Insurance Cohort Data (NHICD), which includes information about ∼1 million patients. This information was obtained from a random sample that was stratified according to age, sex, region, health insurance type, income decile, and individual total medical costs in 2002; the participants were reassessed and followed until 2013. The NHICD includes unique anonymous numbers for each patient as well as their age, sex, type of insurance, diagnoses according to the International Classification of Diseases (ICD-10), medical costs claimed, prescription drugs, and medical history. Moreover, these unique anonymous numbers are linked to mortality information, which was obtained from the Korean National Statistical Office. The Institutional Review Board (IRB) of the Graduate School of Public Health at Yonsei University approved use of these data as well as the study design (IRB approval number: 2-1040939-AB-N-01-2014-239).

### 2.2 Participants

**Figure 1:**
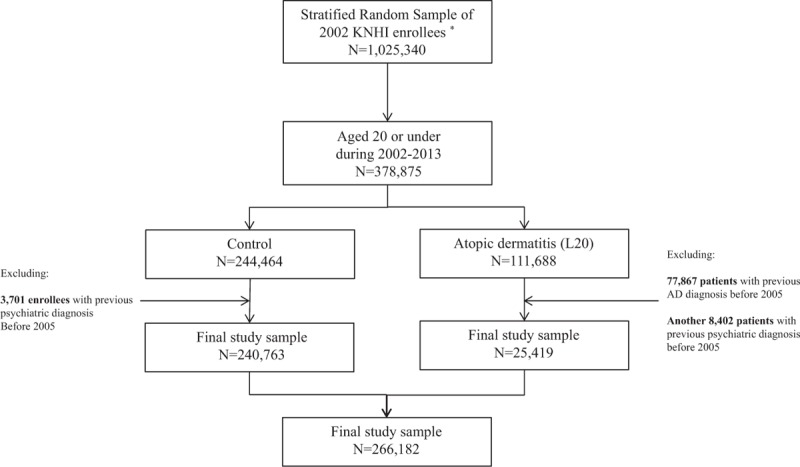
Flowchart showing sample selection. This is a stratified random sampled cohort study with approximately a million subjects. First, we only selected the population aged 20 or under 378,875 patients. Then we divided the AD patients and non-AD control for all the participants. In terms of AD patients, if someone has the previous AD history before 2005 (n=77,867) or psychiatric diagnosis (n = 25,419), then we excluded such subject. Similarly, among the initial control participants, we only excluded the subjects with psychiatric diseases before 2005 (n = 3701). After that, we designed to start the observation from 2005 January. AD = atopic dermatitis.

On December 31, 2002, a total of 1,025,340 NHICD participants were selected for the present study. From this initial population, a cohort of subjects younger than 20 years of age who had been newly diagnosed with AD (ICD-10 code: *L20*) was chosen for the present analyses based on their natural history of AD. To improve the accuracy of the AD diagnosis, the main diagnosis of each patient, but not any concurrent comorbidity observed during their outpatient visits to medical specialists, including dermatologists and pediatricians, was assessed. In total, 111,688 patients with a history of skin symptoms who had been diagnosed with AD by medical specialists during the examination period were selected from the initial population. Because the present study aimed to assess only new cases of AD, the data of 77,867 subjects with a history of AD from 2002 to 2004 were not included in the present analyses. Furthermore, 8402 subjects who had already been diagnosed with a psychiatric disorder prior to the diagnosis of AD were excluded. The present study also included 244,462 carefully selected control subjects aged 20 years of age or younger. Thus, this study included a total of 266,182 subjects, 25,429 of whom were AD patients and 240,763 of whom were control subjects (Fig. 1).

### 2.3 Covariates

We assessed a number of demographic characteristics, including age, sex, and area of residence. Socioeconomic characteristics, such as the income level and the type of medical insurance, were also assessed, and the NHI premium was used as a proxy measure of precise income because it is proportional to monthly income, including earnings and capital gains. The income deciles of the NHI members were categorized into the following 4 groups: low, (first and second deciles), low–middle (third to fifth deciles), high–middle (sixth to eighth deciles), and high (ninth to tenth deciles).

### 2.4 Severity of AD

The severity of AD was measured by reference to the complexity of medications prescribed to treat AD symptoms. Many AD patients with very mild symptoms experience improvements without any medical treatment by using moisturizers and avoiding specific allergens.^[9–11]^ For patients with more severe AD, doctors may prescribe topical or oral medications according to the treatment guidelines for AD.^[1,12]^

Accordingly, all AD patients were categorized based on these operational criteria into the following groups: AD without treatment, AD with topical treatment only, AD with topical treatment plus a systematic steroid, and AD with topical treatment plus a systematic steroid plus a systematic calcineurin inhibitor.

Topical treatment referred to the topical use of any corticosteroid or calcineurin inhibitor, whereas systematic treatment was defined as oral, intramuscular, or intravenous administration of a corticosteroid.

### 2.5 Outcome measures

The primary outcome measure was defined as an outpatient visit to a psychiatric specialist. Although a number of other quality-adjusted outcomes for measuring the mental health of AD patients, such as the Center for Epidemiologic Studies Depression Scale (CESD)-11 or the Dermatology Life Quality Index (DLQI), may be available for hospital-based research, the use of population-based claims data in the present study made it impossible to use such measurements. Additionally, subjects with a history of medical visits for any psychiatric disorder prior to AD onset or the initial follow-up period were excluded from the present analyses.

### 2.6 Statistical analysis

We assessed the demographic characteristics of AD patients at baseline. Continuous variables were expressed as means, standard deviations (SDs), or medians and were compared using Student's *t*-tests or Kruskal–Wallis tests, as appropriate. Baseline categorical variables were expressed as numbers and percentages and compared with a chi-square (*χ*^2^) test. Additionally, the adjusted hazard ratios (HRs) and 95% confidence intervals (CIs) for visits to a psychiatrist were estimated by applying a Cox proportional-hazard regression model. Model fitting was performed using the PHREG command in SAS version 9.3 (SAS Institute Inc.; Cary, NC).

## 3 Results

### 3.1 General characteristics of study subjects

The present study included 266,182 subjects from the target population, and 1,964,831 person-years were examined during the study period (Table 1). Of these subjects, 25,419 were AD patients (9.5%), and 18,290 (6.9%) had a history of visiting a psychiatrist at least once during the follow-up period. AD patients were twice as likely to have visited a psychiatrist as non-AD subjects (*χ*^2^, *P* < 0.001). A comparison of the rates of psychiatric disorders between the groups (psychiatric disorder cases per 100,000 person-years) revealed that the AD patients were 3 times more likely than the control subjects to have a psychiatric disorder (*P* < 0.001).

**Table 1:**
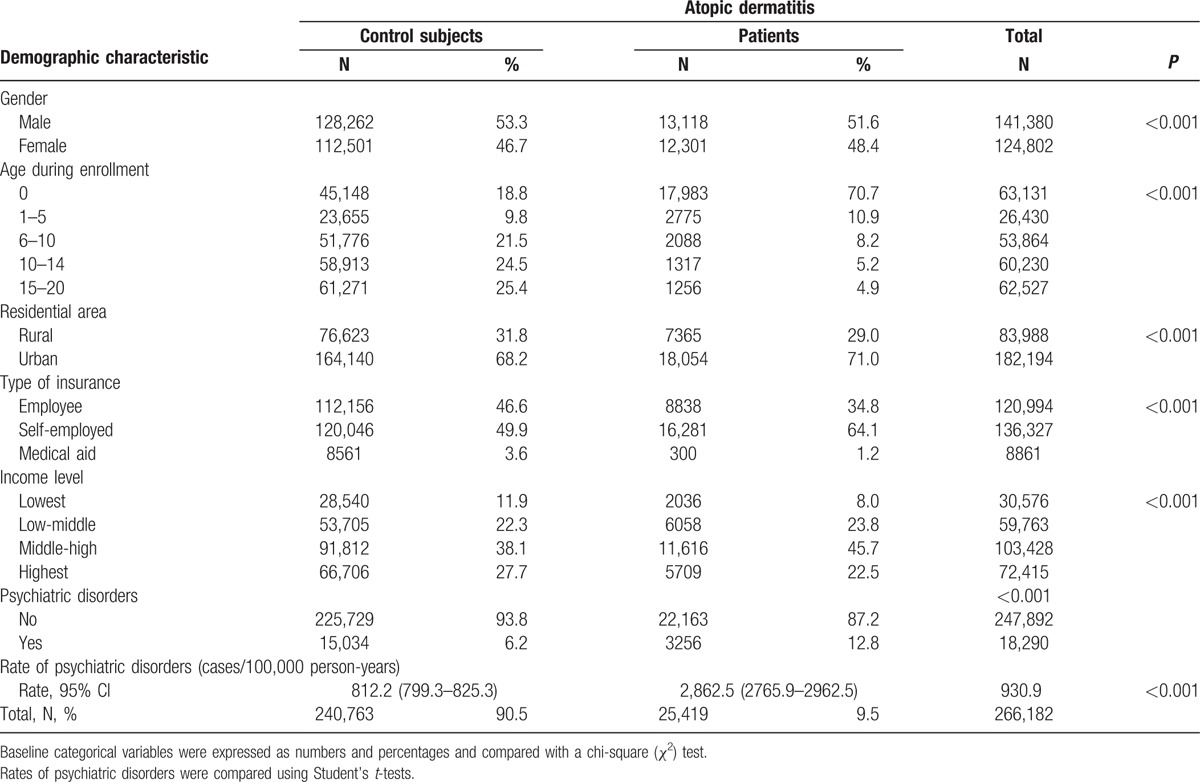
Demographic characteristics of control subjects and patients with atopic dermatitis.

**Figure 2:**
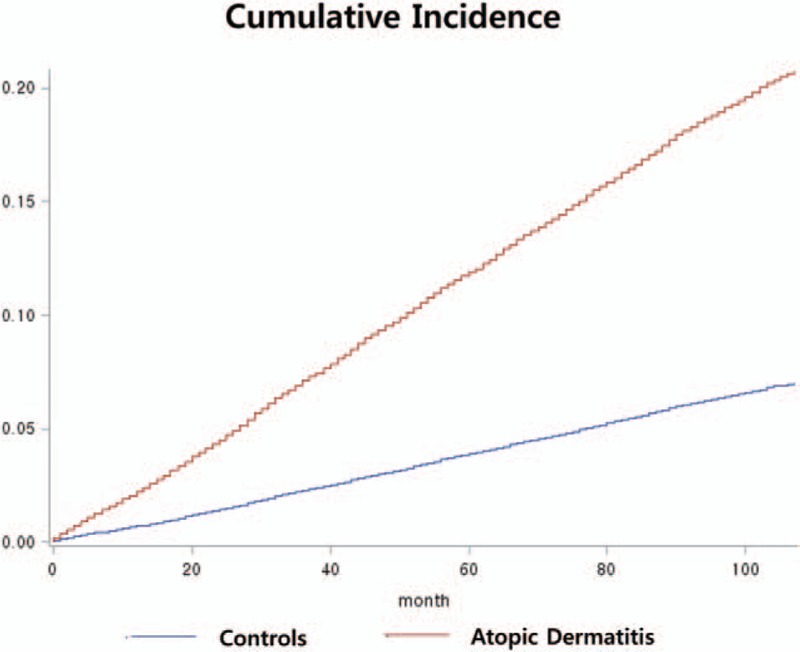
Cumulative incidence of outpatient visits to psychiatrists according to the duration of atopic dermatitis.

Of the AD patients, 41.4% had used only a topical medication, 37.9% did not use a specific medication, and 17.4% had a history of systemic steroidal use (Table 2). An analysis of the history of psychiatric visits revealed that the total AD patient group (all treatments, including those prescribed a systematic calcineurin inhibitor) was 3 times more likely to have experienced psychological problems than was the AD group who had not received treatment (*P* < 0.001). An unadjusted Kaplan–Meier analysis using the log-rank test also revealed that AD patients had a significantly greater chance of visiting a psychiatrist (*P* < 0.001; Fig. 2).

**Table 2:**
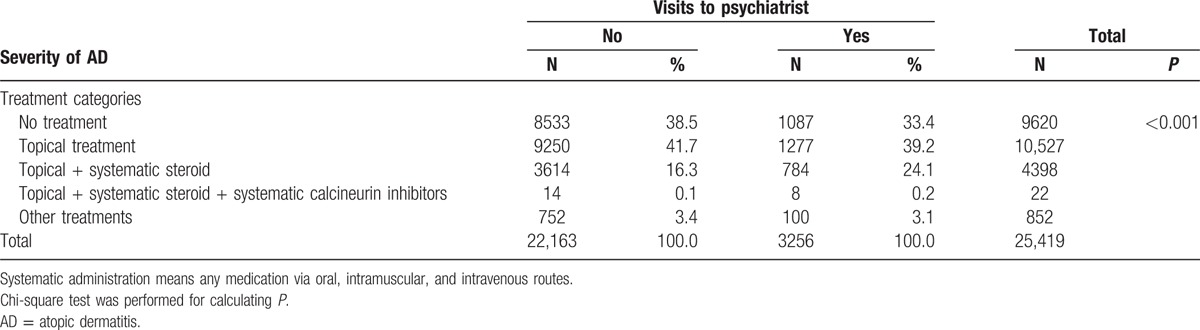
Severity of atopic dermatitis and outpatient visits to psychiatrists.

### 3.2 Multivariate analysis of the HR for visiting a psychiatrist

**Figure 3:**
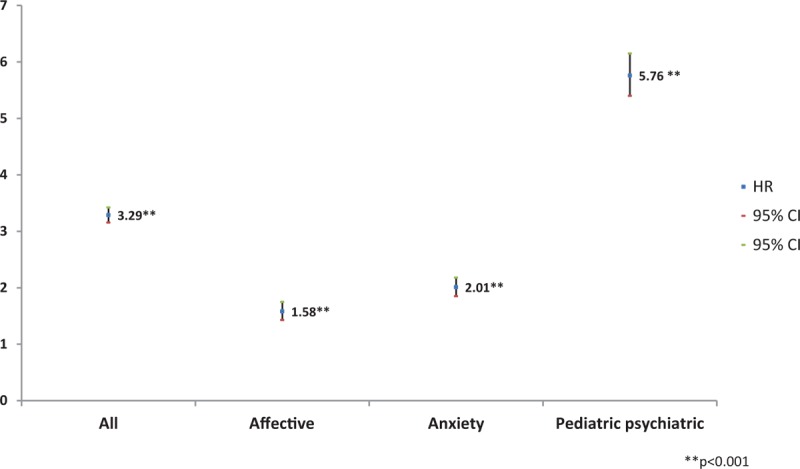
Adjusted HRs for outpatient visits to psychiatrists by patients with atopic dermatitis. We adjusted for gender, age group, residential area, type of insurance, and income level. We determined HRs by subgroup. CI = confidence interval, HRs = hazard ratios.

Patients with AD had a significantly increased adjusted HR (adjusted HR: 3.29, 95% CI: 3.16–3.42) for psychiatric visits for all psychiatric disorders (including affective, anxiety, and pediatric disorders) compared with the controls (Fig. 3). In particular, the likelihood of visiting a psychiatrist for a pediatric psychiatric disorder was ∼5 to 6 times higher for AD patients compared with those without AD (adjusted HR: 5.76, 95% CI: 5.40–6.15).

Compared with subjects without a history of AD, patients with a medical history of AD only, and who were not specifically treated, had a higher adjusted HR for psychiatric visits (adjusted HR: 3.70, 95% CI: 3.43–3.98; Table 3). Additionally, the adjusted HRs significantly increased with the complexity of the AD medication regimen. AD patients who had received only a topical treatment showed had an increased HR for psychiatric visits (adjusted HR: 4.50, 95% CI: 4.21–4.80). Moreover, AD patients who had received a topical treatment plus a systematic steroid (adjusted HR: 4.88, 95% CI: 4.51–5.27) and AD patients who had received a topical treatment plus a systematic steroid plus a systematic calcineurin inhibitor (adjusted HR: 9.56, 95% CI: 4.29–21.28) were associated with even greater increases in the HRs for psychiatric visits.

**Table 3:**
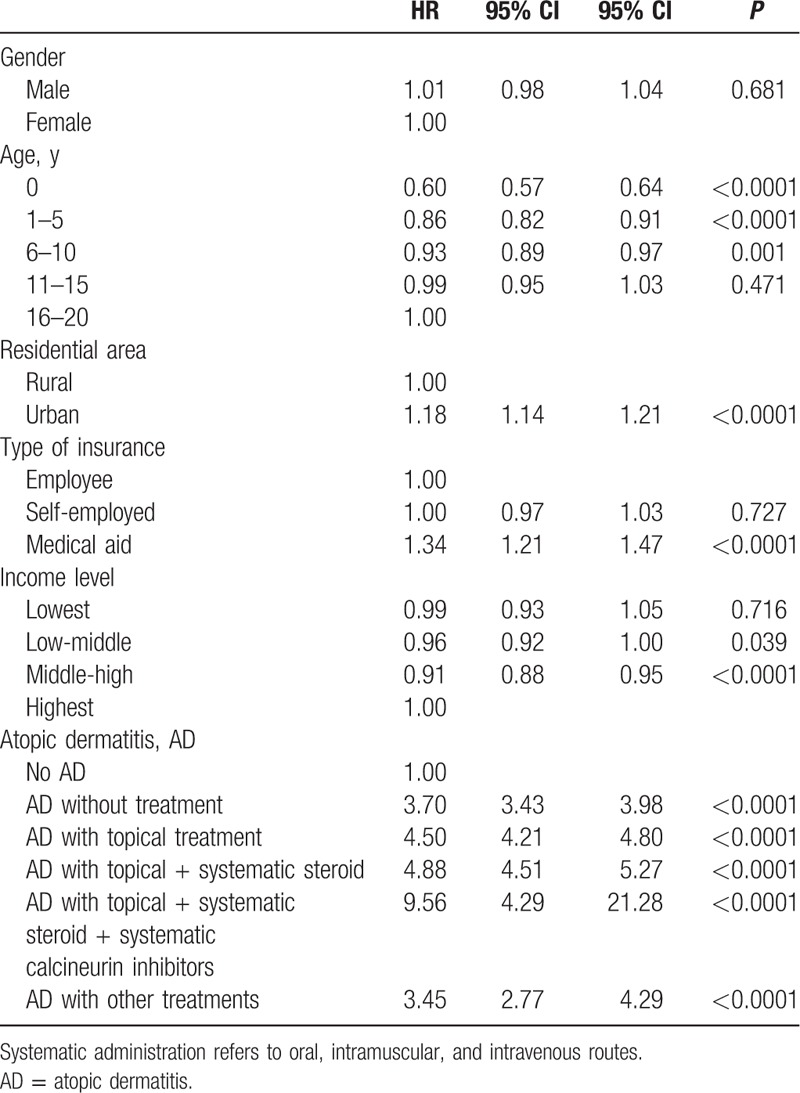
Adjusted hazard ratios for outpatient visits to psychiatrists (all psychiatric disorders).

Subgroup analysis based on age showed that young AD patients, especially those aged 6 to 10 years, had increased adjusted HRs for visiting a psychiatric clinic (Supplementary Figure 1) as the variety of their medications increased. In addition, AD patients in both areas showed a gradually increasing tendency toward a higher HR as the complexity of their medication regimen increased (Supplementary Figure 2).

## 4 Discussion

Several studies have investigated the relationship between AD and psychiatric disorders, particularly depressive mood disorders.^[7,13–15]^ Similar to these studies, the present investigation using a large sample size and a longitudinal design supported the hypothesis that there is a positive association between AD and an increased risk of affective, anxiety, and/or pediatric psychiatric disorders. Additionally, this study also evaluated the severity of AD and found a dose-dependent relationship between the severity of this disease and the likelihood of developing a psychiatric disorder. Based on the most recent AD treatment guidelines from Europe and North America,^[12,16]^ it is reasonable to classify the severity of AD according to the complexity of the medication regimen because topical and systematic calcineurin inhibitors are both regarded as standard treatments for this disorder.

Although the exact mechanisms underlying this relationship require further investigation, there are several possible factors that may link AD with the development of psychiatric disorders or other mental health burdens.

First, many AD patients complain of sleep disturbance associated with aggravated symptoms and have significantly reduced sleep efficiency, longer sleep-onset latency, a greater degree of sleep fragmentation, and less nonrapid eye movement sleep.^[13,15,17–19]^ More specifically, higher total and/or allergen-specific immunoglobulin E (IgE) levels are associated with lower nocturnal melatonin secretion, which can disturb sleep induction and maintenance.^[17]^ Additionally, brain-derived neurotrophic factor (BDNF) and Substance P are related to disease severity, quality of life, and nocturnal scratching in AD patients.^[20]^ These types of stress-induced biological factors can interfere with the skin barrier system, including the epidermis, and aggravate nocturnal pruritus.^[21]^ Thus, psychological stress and AD symptoms appear to form a vicious circle because the sleep disturbances experienced by AD patients result in inappropriate sleep quality and quantity and increase mental health problems, including psychiatric disorders.^[14,22–24]^

Second, the negative cosmetic aspects of AD affect self-esteem and impair interpersonal relationships. Patients with moderate-to-severe AD typically suffer from oozing and erythematous skin patches with scales during the acute stage of the disease.^[3,18]^ Moreover, the skin lesions are transformed during the transition from the acute to the chronic phase, and the lichenification and dark-reddish skin colors may result in significant levels of stress in patients’ social lives.^[25]^ Thus, AD is associated with high levels of stigmatization, social withdrawal, anxiety, and depression among patients and may affect their careers.^[13,15,26]^ AD-related perceptions of stigma are of greater importance than more general psychological factors when predicting AD-related quality of life, such as the experience of depression.^[27,28]^ Moreover, children and adolescents with AD repeatedly experience feelings of social isolation, peer-group rejection, teasing, and bullying, which may lead to a loss of confidence, mood changes, and/or depression.^[29,30]^ This is important, because these kinds of emotional experiences can impair concentration during class; in fact, students with severe AD are often absent from school.^[31,32]^ Such factors ultimately have a negative effect on education and can lead to children becoming withdrawn^[33]^; it may also increase the likelihood that they will exhibit difficult behavior or even lead to the development of pediatric and adolescent psychiatric disorders.^[7,13,26]^ Thus, the cosmetic discomfort of AD is significantly related to mental health.

Third, AD and psychiatric disorders may share pathogenic factors related to genetic susceptibility and inflammation. In terms of immunological pathways, several studies have shown that pro-inflammatory cytokines secreted by eczematous inflammation and some stress hormones, including oxytocin glucocorticoid from epidermal keratinocytes, penetrate the blood–brain barrier (BBB) and activate neuropathogenic mechanisms related to emotional control.^[34–38]^ In this context, it is possible that there is an initial sequential activation of AD via the actions of inflammatory cytokines and hormones and that a psychiatric disorder may develop later. However, because there is controversy regarding the bidirectional relationship between psychological factors and AD onset,^[39]^ further investigation is needed to elucidate the precise mechanisms underlying AD and mental illness. The present study has several limitations that should be addressed.

First, the NHICD does not include detailed medical information such as disease severity, personal lifestyle, perceived stress, or other environmental factors. However, this study attempted to adjust for all available socioeconomic factors, including household income, insurance type, and residential area, as well as for demographic factors, such as age, gender, and duration of study participation.

Second, the database is not linked to family members to protect personal privacy; thus, it was impossible to determine or adjust for family history of allergic diseases, which is one of the most important factors in the severity of AD. On the other hand, because the complexity of prescribed medications was utilized as a proxy indicator for disease severity, we at least partially accounted for this limitation.

Third, the incidence of AD may have been overestimated, whereas the rates of psychiatric disorders may have been underestimated. Although only AD patients diagnosed by board-certified dermatologists and pediatricians were included in the present study based on the hypothesis that specialists are able to correctly diagnose AD, it is sometimes difficult to distinguish AD from other eczematous disorders. However, as the prevalence of AD in the study population was 9.5%, this may reflect the epidemiologic characteristics of AD.

Fourth, the psychiatric visits do not mean the psychiatric problems. Some patients might visit the clinics to consult their insomnia without regular medication. However, we could not distinguish this kind of patients from participants. To figure out, we need some further investigation with health record review.

Fifth, the other AD treatments were not considered in this study. It is excluded that some other systematic treatments including azathioprine, mycophenolate, and methotrexate and phototherapy for AD. Because some of these treatments were not covered by NHI, we could not exactly consider them.

Finally, although this study included broad CIs in the statistical analyses, the relatively small sample size of 25,419 AD patients may limit the generalizability of the results. Further, clinical studies that involve matching with real medical records are required to validate the present findings.

## 5 Conclusions

In conclusion, this population-based longitudinal cohort study demonstrated that child and adolescent AD patients have a higher risk of developing psychiatric disorders, and this risk increases as the complexity of their medication regimen increases. Moreover, screening for early stress and psychiatric disorders based on the diversity of AD medications may be the easiest and most efficacious method with which to identify at-risk populations while accruing the lowest administrative cost. However, since we could not adjust psychological stress, the further study should be needed for the association between AD onset and developing psychiatric disorders.

## Acknowledgments

This manuscript was reviewed by Textcheck, a professional English-language editing service. We also thank the members of the Department of Dermatology and of the Cutaneous Biology Center at Yonsei University for their detailed feedback on this manuscript.

## Supplementary Material

Supplemental Digital Content
