# Supplementary material for: Psychiatry outpatient visits by atopic dermatitis patients varying in the complexity of their prescriptions: A nationwide cohort study conducted from 2005 to 2013
Source: Medicine (Baltimore). 2016 Dec 9;95(49):e5411. doi: 10.1097/MD.0000000000005411 (PMC5265989; doi:10.1097/MD.0000000000005411)

**Supplementary materials**

**Supplementary Figure 1.** Age-specific analysis in 5-year intervals. (A) 0–5 years; (B) 6–10 years; (C) 11–15 years; (D) 16–20 years.


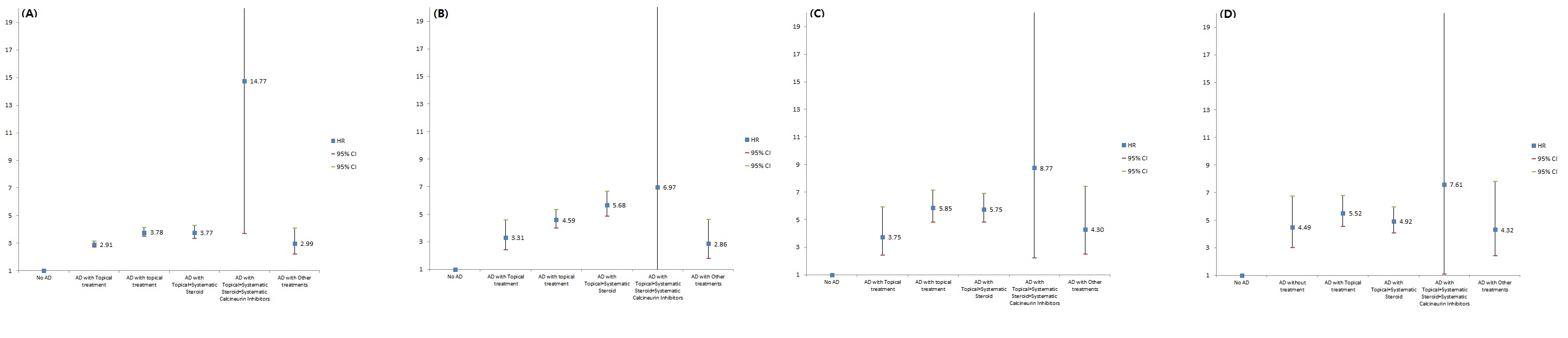


**Supplementary Figure 2.** Living area-specific analysis (urban and rural.) (A) urban; (B) rural.

The English in this document has been checked by at least two professional editors, both native speakers of English. For a certificate, please see:

http://www.textcheck.com/certificate/VVaLgT


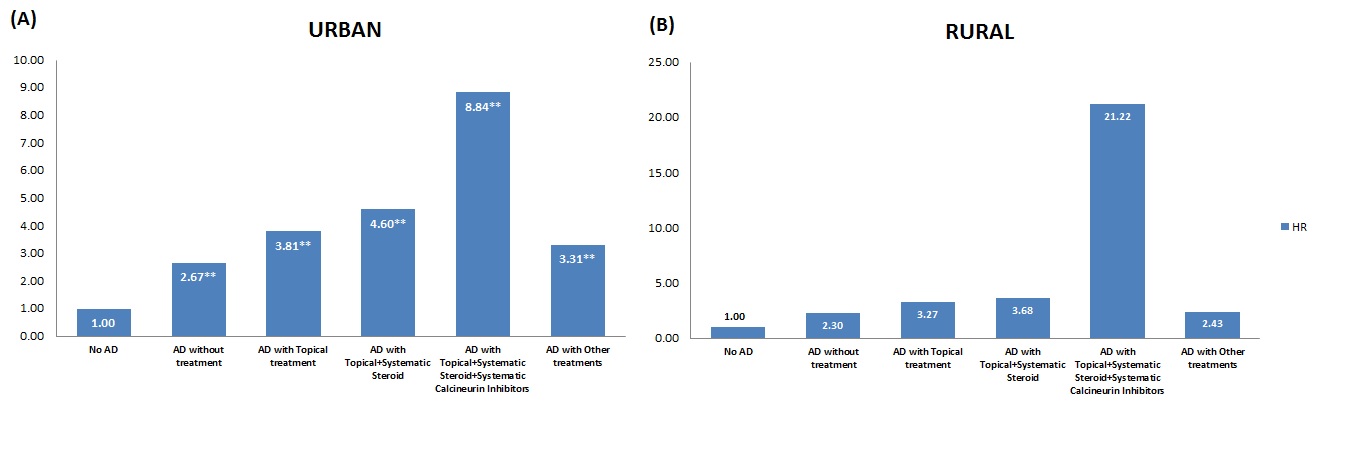

Supplement: Supplemental Digital Content [file medi-95-e5411-s001.doc]
